# Role of Counterions in the Adsorption and Micellization Behavior of 1:1 Ionic Surfactants at Fluid Interfaces—Demonstrated by the Standard Amphiphile System of Alkali Perfluoro-*n*-octanoates

**DOI:** 10.1021/acs.langmuir.1c00527

**Published:** 2022-01-07

**Authors:** Klaus Lunkenheimer, Dietrich Prescher, Katrina Geggel

**Affiliations:** † Max-Planck-Institut für Kolloid- und Grenzflächenforschung, Am Mühlenberg 1, Potsdam D-14476, Germany

## Abstract

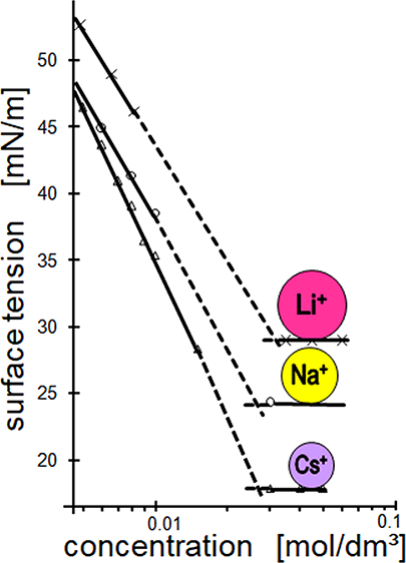

In our latest communication, we proved experimentally that the ionic surfactant’s surface excess is exclusively determined by the size of the hydrated counterion.[Lunkenheimer, Langmuir*,*2017, 33, 10216−1022410.1021/acs.langmuir.7b00786]28925711. However, at this stage of research, we were unable to decide whether this does only hold for the two or three lightest ions of lithium, sodium, and potassium, respectively. Alternatively, we could also consider the surface excess of the heavier hydrated alkali ions of potassium, rubidium, and cesium, having practically identical ion size, as being determined by the cross-sectional area of the related anionic extended chain residue. The latter assumption has represented state of art. Searching for reliable experimental results on the effect of the heavier counterions on the boundary layer, we have extended investigations to the amphiphiles’ solutions of concentrations above the critical concentration of micelle formation (cmc).We provided evidence that the super-micellar solutions’ equilibrium surface tension will remain constant provided the required conditions are followed. The related σ_cmc_-value represents a parameter characteristic of the ionic surfactant’s adsorption and micellization behavior. Evaluating the amphiphile’s surface excess obtained from adsorption as a function of the related amphiphile’s σ_cmc_-value enables you to calculate the radius of the hydrated counterion valid in sub- and super-micellar solution likewise. The σ_cmc_-value is directly proportional to the counterion’s diameter concerned. Taking additionally into account the radii of naked ions known from crystal research, we succeeded in exactly discriminating the hydrated alkali ions’ size from each other. There is a distinct sequence of hydration radii in absolute scale following the inequality, Li^+^ > Na^+^ > K^+^ > (NH_4_)^+^ > Rb^+^ > Cs^+^. Therefore, we have to extend our model of counterion effectiveness put forward in our previous communication. It represents a general principle of the counterion effect.

## Introduction

**Figure 1:**
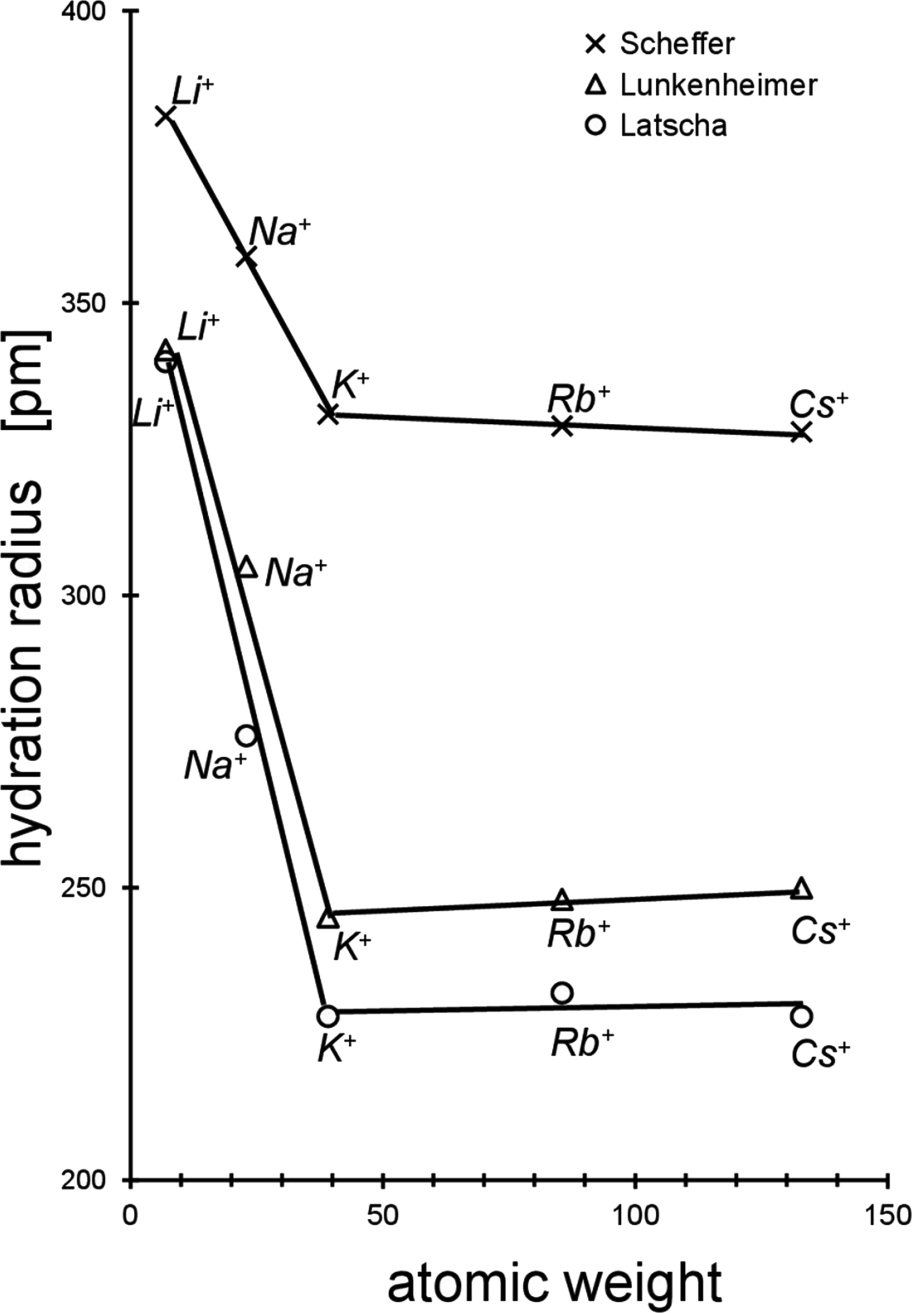
Radii of hydrated counterions as a function of their atomic weight. The data of Lunkenheimer et al. (triangles) are the ones calculated from the surface excess Γ_∞_ of the experimental σ_e_ vs log *c* isotherm at a sub-micellar concentration.^2^

In refs (1) and (2), we have reported on the behavior of counterions in adsorption layers of 1:1 ionic alkali perfluoro-*n*-alkanoates at the air/water interface. The latter class of surfactants is convenient in particular for such kind of study insofar as its adsorption obeys ideal surface behavior and these amphiphiles are not subjected to degradation in aqueous solution. In ref (2), we could establish proof for the pseudo-nonionic system of alkali perfluoro-*n*-octanoates in which their counterions are discretely bound to the related ionic head groups in a non-random manner. Due to this, the minimal cross-sectional area of the adsorbed anionic amphiphile will exclusively be determined by the geometrical size of the hydrated cation. However, we could provide reliable evidence for it only for the most important alkali cations, i.e., for the lighter ones of lithium (Li^+^), sodium (Na^+^), and potassium (K^+^). These findings have qualitatively been in line with data of textbooks^3,4^ and our latest ones.^2^ Is it really true that the hydration radii of the heavier alkali ions are practically equal? This, in turn, would mean that their surface excess is determined by the amphiphile’s anionic residue, but any peculiarity of its counterion remains negligible. Looking for a reference state by means of which we can decide about the apparent contradictions between the lighter and the heavier counterions’ behavior, we remembered Mendeleev’s system of Periodic Table of the Elements,^5^ the 150th anniversary of its issue we have celebrated just now. The ingenious inventor Mendeleev said that the chemical character of the elements changes periodically in a way that chemically related elements are assembled in eight separate main groups. With respect to the question raised above, it is important to underline that Mendeleev’s prediction did not only concern the properties of the elements themselves but also the ones of their compounds. The properties of the compounds should then alter as a function of their atomic weight too. Thus, for example, the naked, crystal ion radii of alkali cations, which are exactly known,^6−8^ do continuously increase from the lightest atom of Li^+^ to the heaviest one of francium Fr^+^. However, the hydration radii of refs (3, 4), and (2) having so far been considered to be true, as shown by Figure 1, reveal a strange discrepancy. This feature is prone to make the reader believe that the amphiphiles’ adsorption is characterized by its anionic extended chain residue, according to the state of art, but the ones of the biggest counterion will represent a noteworthy exception to the general rule.

However, having this result would contradict Mendeleev’s law.

Before going into detail, we need to emphasize on the important experimental condition that is required to draw any reliable conclusions on the amphiphiles’ true adsorption properties. During the experimental investigations, we conscientiously took into account the fact that the surfactant solutions did possess the particular grade of “surface-chemical purity”.^9,10^ Due to the latter circumstance, we in our previous experimental investigations had purified the related stock solutions well below the critical concentration of micelle formation (cmc). It appears that these conditions have also been straightforward in detecting the special role of counterions in adsorption.^2^ During these previous investigations, our concern was focused only at the range of sub-micellar concentrations, *c* < cmc.

After we had detected that the counterion size will become of utmost importance in the adsorption of pseudo-nonionic amphiphile systems, but still being bothered with the strange cation hydration radii feature, illustrated in Figure 1, we in a subsequent approach have measured equilibrium surface tension of the said alkali perfluoro-*n*-octanoates at micellar concentrations, *c* ≥ cmc, too. There, we observed a striking phenomenon. First, the equilibrium surface tension value at and above the cmc represents a discrete constant. This holds for all alkali perfluoro-*n*-octanoate surfactants investigated. Second, the corresponding σ_cmc_-values differ from each other tremendously, up to 11 mN/m. Third, although the differences in the σ_cmc_-values are comparatively great for the bigger hydrated counterions only, the hydration size of the heaviest cations rubidium (Rb^+^) and cesium (Cs^+^) can obviously well be discriminated by the related σ_cmc_-values. As mentioned above, cf. Figure 1, the hydration radii of these counterions have so far been assumed to be hardly discernible.^3,4,11^

In ref (2), we came to the conclusion that you must not apply exceptional hypothesized properties other than the ones valid in basic physical chemistry to describe counterion effects in adsorption.

Thus, reminding the findings that the common adsorption parameters of the alkali perfluoro-*n*-octanoates are stable and reliable,^1,2^ we asked what the detailed physicochemical meaning of the different σ_cmc_-values is due to and whether it would be possible to determine the absolute size of the hydration sphere of the related alkali counterions solely by their surface tension data without regard to the hydration data received from other bulk methods. If this approach was successful, the resulting hydration radii would be the true ones encountered under experimental conditions in the boundary as well as in the bulk phase.

## Experimental Section

### Synthesis

Details of the alkali perfluorooctanoates’ synthesis are given in refs (1) and (2). Principally, they were prepared from perfluoro-*n*-octanoic acid (99% *n*-isomer, ABCR GmbH & Co., Karlsruhe). The salts were generally prepared from the perfluoro-*n*-octanoic acid by neutralization in aqueous solutions with equivalent amounts of alkaline carbonates or with alkaline hydroxides, or with ammonium hydroxide with purity of the highest analytical grade available (“as received”).^12−14^ The obtained alkali perfluorooctanoates were purified by repeated recrystallization. Their purity was checked by paper chromatography (PC) according the method described for alkanesulfonates.^14^

### Purity

During our previous work on counterion effects in adsorption, we have generally carefully taken into consideration that the surfactant solutions concerned did have the particular grade of “surface-chemical purity” (scp). This was confirmed by applying special measures to upgrade the “as received” (ar) stock solution into its scp-state.^9,10^

The requirement for “surface-chemical purity” is especially important for the application of perfluorinated surfactants since the electrochemical perfluorination process does not provide good yields,^15−17^ no better than 45–50% at maximum according to ref (15). Fortunately, recently, a new procedure to produce perfluoroalkanoic acids with better yield has been applied.^17^

### Experimental Determination of Correct Surface Tension Values σ_cmc_ at the Critical Concentration of Micelle Formation *c* ≥ cmc

Generally, the concentration of the ar-stock solutions is applied at *c* < cmc to avoid the impure components becoming hosted within micelles and thus “hidden” from the boundary layer. Searching for exact equilibrium surface tension values of surfactant solutions at the critical value of micelle formation, σ_cmc_, and/or at concentration *c* ≥ cmc, you will find a dissatisfying result in the literature. It seems as if a general characteristic behavior of the equilibrium surface tension value σ_cmc_(*c*) at concentrations *c* ≥ cmc could not be established, irrespective of the surfactants’ chemical nature, nonionic or ionic. As these references are too voluminous, we do not refer to any of them especially. If we confine on the family of perfluoro amphiphiles, the function σ_cmc_(*c*) seems to be approximately constant in most cases.^18−20^ However, you will meet quite a few contradictory results, nevertheless, depending on the reference given.^15,21,22^ With respect to the alkali perfluorooctanoate compounds, reported on here, the results of the most recent study ref (22) on the surface tension behavior above cmc appears rather strange. There are two inflection points of the σ vs log *c* isotherm at the sub-micellar concentrations and a maximum within the experimental functionality of σ_cmc_(*c*) at concentrations above cmc. Thus, unfortunately, it has so far not been possible to attribute a confident σ_cmc_-value characteristic at least to one single compound, such as the most common standard alkali perfluoro-*n*-alkanoate, i.e., that of sodium perfluoro-*n*-octanoate.

It was already Elworthy and Mysels who had detected with the “classical” hydrocarbon surfactant system sodium dodecylsulfate (SDS) that the usually observed minimum of its σ_e_ vs log *c* isotherm at concentrations *c* ≅ cmc disappeared by further special, thorough purification by applying foaming.^23^ Later, we have shown that any surfactant system when applied in the purity grade “as received” (ar) usually contains traces of surface-active contaminants possessing comparatively stronger surface activity than that of the main surfactant, which will effectively falsify the latter’s surface properties. We have found that it is these contaminants that also cause minima in the σ_e_ vs log *c* isotherms of micelle-forming amphiphiles in the neighborhood of their cmc-values. Lunkenheimer and Miller have derived a criterion to prove the mandatory grade of “surface-chemical purity” at sub-micellar concentrations *c* < cmc.^9^ This criterion is related to the rising surface tension after cyclic operations of compressing and sucking of the surfactant solutions’ surface.^10,24^

This criterion can be applied at concentrations above cmc too. However, we then have to take into account that the state of surface-chemical purity does hold for these particular concentrations *c* ≥ cmc only. It is important to realize that contrary to sub-micellar conditions when the effect of contamination becomes maximal at the concentration of the stock solution, at super-micellar concentrations, the purification procedure has to be performed for each concentration separately because the effect of impurity on the surface layer becomes maximal exactly at the cmc.^23^ It is detectable by a minimum in the σ_e_ vs log *c* isotherm in the neighborhood of the cmc, including concentrations *c* ≥ cmc, usually observed with surfactant solutions of “as received” quality, cf. above.

If we observe that the surface tension value σ_cmc_ for which the required scp quality was proven to remain constant at higher super-micellar concentrations *c* ≥ cmc too, it means that the adsorption layers of these solutions will also possess the required scp-grade. In this case,
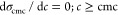
1

To ensure that we gain the true σ_cmc_-value, we have performed surface-chemical purification at concentrations somewhat greater than cmc, usually at twice or threefold cmc. We have applied the purification apparatus described in refs (10) and (24). In case the state fulfilling the condition dσ_e_/dj = constant, i. e.,
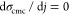
2the correct σ_cmc_-value of the alkali perfluorooctanoate surfactant will be gained.

## Results and Discussion

By this investigation, we proved that the absence of intermingling trace impurities at bulk concentrations at and above the critical concentration of micelle formation will result in a constant, characteristic equilibrium surface tension value σ_cmc_. Thus, if this σ_cmc_-value was verified by eq 2, it would be evident that it represents the true numerical surface parameter belonging to micellar and super-micellar conditions *c* ≥ cmc, respectively. Applying the symbol σ_cmc_ in what follows it will always refer to this experimentally verified true equilibrium surface tension value at cmc ≤ *c* ≤ 5 × cmc.

**Figure 2:**
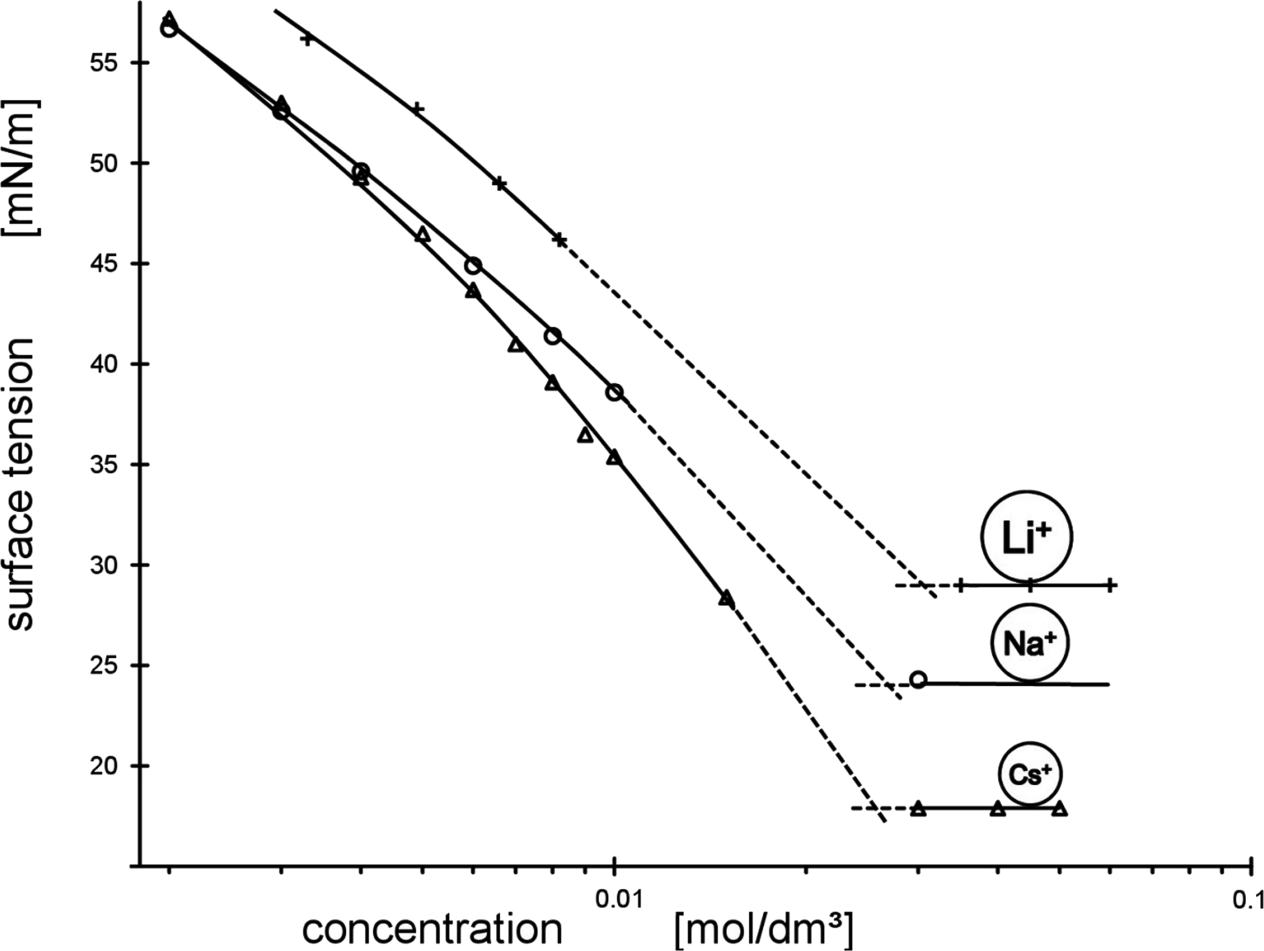
Part of the three extreme alkali perfluorooctanoates’ equilibrium surface tension (σ_e_) versus bulk concentration (*c*) isotherms in the neighborhood of the related critical concentration of micelle formation (cmc). The dotted lines mark the extrapolated course calculated by the adsorption isotherm. The intersection with the constant σ_cmc_-value marks the cmc value each. The cross-sectional areas of the three extreme cation radii of lithium, sodium, and cesium, respectively, are added using empty circles (in scale). In this figure, there is only one measuring σ_cmc_-value of sodium perfluorooctanoate. However, it was proven in Figure 1 of ref (1) that this is the correct one.

Taken by surprise, there has been an overwhelming effect of the counterion on the σ_cmc_-value concerned. Figure 2 shows part of the three alkali perfluorooctanoates’ σ_e_ vs log *c* isotherms in the neighborhood of the related cmc, taken from ref (2). As a matter of fact, the Δσ_cmc_ difference between Li^+^- and K^+^-pf-perfluorooctanoate amounts to about 10 mN/m. The overall difference of the extreme σ_cmc_-values between Li^+^- and Cs^+^- pf-octanoate amounts to 11.3 mN/m. If we relate it to the measuring accuracy of the ring tensiometer applied, amounting to ∓0.1 mN/m, this finding will prove an exceptional measuring sensitivity of the solutions’ surface tension to the effect of counterion.

Following the literature, there are various proposals explaining the cause for the counterion effects. The most conceivable ones are due to their calorimetric and geometric nature, respectively. To look for the true one, we have investigated the former by calculating the standard free energy of micellization of these alkali perfluorooctanoates:
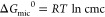
3

Evidently, the various cmc-values do not differ from each other much (cf. Figure 2). The cmc-values were calculated by extrapolating the functional course of the experimental σ_e_ vs log *c* isotherms toward their related constant σ_cmc_-values. Thus, we retrieved the formers from sub-micellar solutions for all of which surface-chemical purity was guaranteed. The resulting intersection point of the best-fit function provides the concentration of micelle formation, cmc.

**Figure 3:**
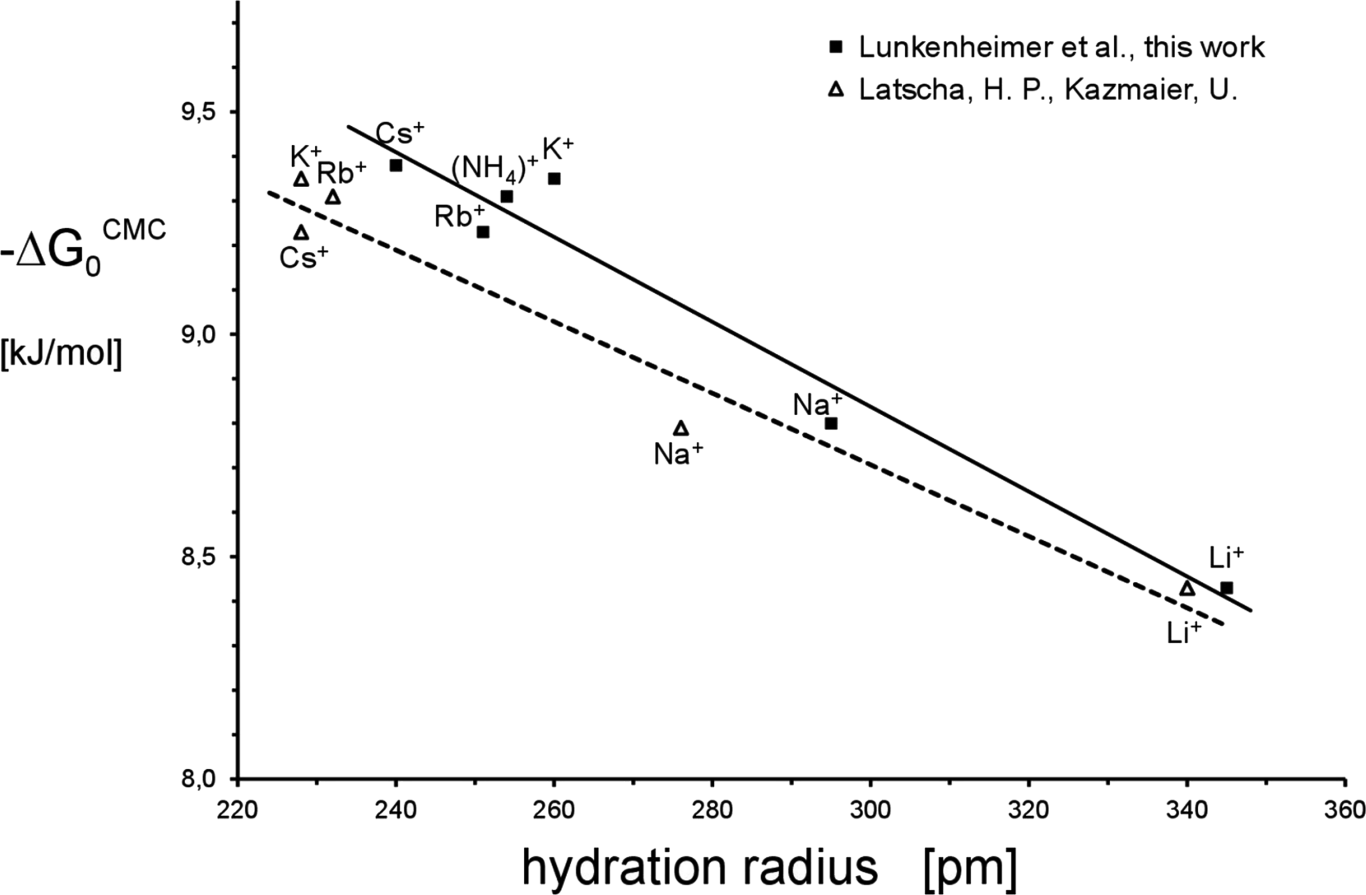
Standard free energy of micellization Δ*G*_mic_^0^ of lithium-, sodium-, potassium-, rubidium-, cesium-, and ammonium-perfluorooctanoates as a function of the cations’ hydration radius (from the literature^3^ and Lunkenheimer et al.^1,2^ [this communication]).

Considering the Δ*G*_mic_^0^ values as a function of their related counterion radii taken from refs (2) and (3) reveals distinct dependency for all of the five amphiphiles from lithium to cesium perfluorooctanoate (Figure 3). The standard free energy of micellization is inversely proportional to the square of the counterion radius. The best-fit quality is excellent as characterized by *r*^2^ = 0.993. If we use the (*r*^+^)_aq_-values of our approach (see below), the matching quality deteriorates somewhat to *r*^2^ = 0.953. However, it remains a very good best-fit, nevertheless, simply by applying the plain linear equation of *y* = *a* + *bx*. Its maximal error of Δ*G*_mic_^0^ amounts to 1.5% only.

Favorable in this case, the correct values of the heaviest ions together with that of the ammonium ion are included. Taking into account that the overall change in Δ*G*_mic_^0^ is only about 10%, this result is remarkable. It proves that there is a distinct, measurable effect of counterions on the thermodynamic properties of micellization, although a comparatively weak one only. The above data on hydration radius were taken from ref (3). Applying the related data from ref (4) results in a similar best-fit. However, there is a considerable shift of the Δ*G*_mic_^0^ versus (*r*^+^)_aq_ relationship along the abscissa due to the much greater radii of hydration in ref (4). In addition the slope of the dependency dΔ*G*_mic_^0^/d(*r*^+^)_aq_ gets considerably steeper due to the much smaller maximal difference between the biggest and the smallest counterion radii. This difference amounts to 112 pm in ref (3) but only to 54 pm in ref (4).

Hence, the counterion effect occurs regularly well in line with steadily altering ion radii. Moreover, the trend observed is compatible with basic assumptions on surface thermodynamics.

The change of standard free energy of micellization on the size of the counterion hydration diameters takes place the strongest with the lighter cations. In terms of surface thermodynamics, it means that the bigger counterion’s hydration shell will go in line with a somewhat smaller surface activity of the amphiphile. Taking into account the achievement in ref (2) saying that the effect of counterions bound to the surfactant’s anionic residue of a pseudo-nonionic amphiphile resembles that of a nonionic amphiphile’s hydrophilic head group, this result is reasonable. Although all alkali cations are completely hydrophilic in nature, small differences in their hydrophilicity are encountered, nevertheless, by it affecting the corresponding pseudo-nonionic amphiphile’s surface activity, which weighs the ratio of hydrophobicity and hydrophilicity.^25−27^ This phenomenon has been verified by the adsorption behavior of nonionic surfactants whose polar head groups’ hydrophilicity had been discretely changed. For example, the slight change in hydrophilicity may be brought about by definite ethylene or propylene oxide groups^28,29^ and/or by substituting the polar nonionic head group for hydrophobic residues of different sizes.^30,31^

**Figure 4:**
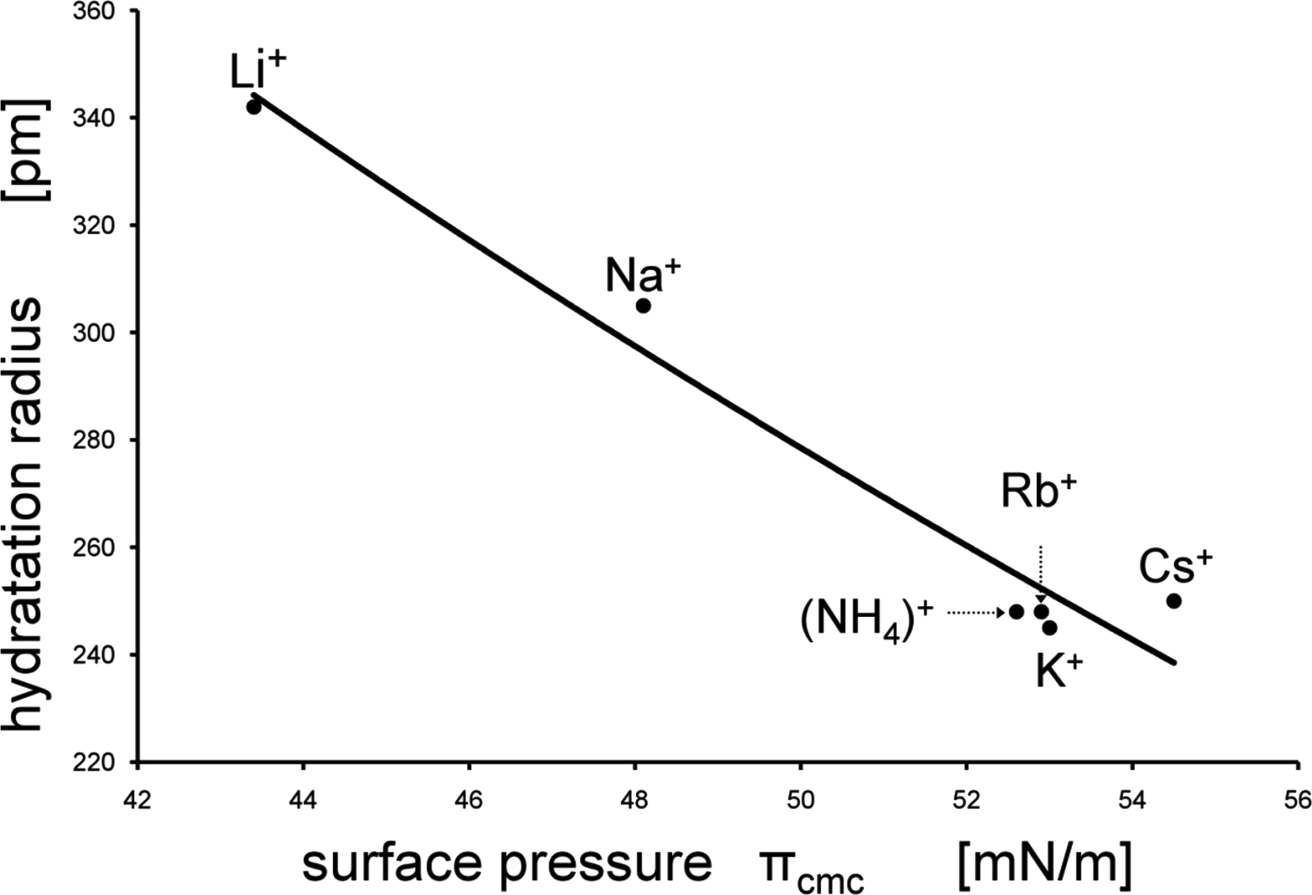
Hydration radius *r*^+^_aq_ as a function of measured surface pressure, π_cmc_-values.

All in all, however, the caloric effect of counterion size remains small. Thus, the alkali counterions’ great effect on the parameter of σ_cmc_ cannot be explained by a small thermodynamic difference of their standard free energy of micellization. Therefore, it seems reasonable to conclude that the characteristic big differences in the σ_cmc_-values originate genuinely from the cations’ geometrical properties, i.e., from their hydration radii, as we have found already for the adsorption parameters in ref (2). Searching for the true meaning of the parameter σ_cmc_, we again draw your attention to the sensitivity of surface tension measurement. It shows that our approach ought to get especially favorable to pursue the reason of the counterion effect. As mentioned above, the maximal difference between the σ_cmc_-values of Li^+^- and Cs^+^-perfluorooctanoate amounts to Δσ_cmc_ = 11.1 mN/m. Relating it to the measuring accuracy of the tensiometer of about ∓0.1 mN/m, we could expect an accuracy of about 1% if we used investigations on surface tension to determine counterion hydration. It indicates the preferential suitability of surface tension measurement for this particular aim. In our latest work, we have derived the cations’ hydration radii by evaluating the experimental adsorption isotherm σ_e_ vs log *c* measured at sub-micellar concentrations. Now, we do relate surface excess Γ to micellar solutions additionally. From the adsorption isotherm, we know that surface excess is maximal at the cmc. Hence, Γ_cmc_ = Γ_∞_ should hold. In other words, if we plot the surface pressure π_cmc_ against the saturation adsorption parameter Γ_∞_ or the corresponding cross-sectional area *A*_min_ and/or the related hydration radius *r*^+^_aq_ of the adsorbed amphiphilic molecule, a reasonable relationship should result. However, following the above experimental finding on the great reliability of the π_cmc_-values, we advantageously consider the surface pressure π_cmc_ as an independent variable and calculate now the ions’ hydration radii from the maximal surface excess by (*r*^+^)_aq_(π_cm_) (Figure 4). π_cmc_ = σ_w_ – σ_cmc_, σ_w_ denotes the surface tension of pure water.

The simple relationship
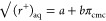
4results in a best-fit quality of *r*^2^ = 0.962, possessing a maximal error in (*r*^+^)_aq_ of 5%.

As a matter of fact, there is a strict relationship between the ions’ hydration radius and the related π_cmc_-values. The most important result of it consists in the fact that by our novel approach, we became able to prove by experiment that there is indeed a successive decrease of the cations’ hydration radii from Li^+^ down to Cs^+^. Comparing these results with those of the textbooks of refs (3) and (4), we first can state that our approach results in a remarkable improvement insofar as the *r*^+^_aq_-value of the heaviest ion cesium is indeed distinctly lower than those of the related ions of Rb^+^ and K^+^. Unlike our findings, the data of refs (3) and (4) have in common the fact that the hydration radii of the cations K^+^, Rb^+^, and Cs^+^ are practically identical. This result underlines the suggestion mentioned in the introduction that it is to be expected that the hydration radii of the cations should decrease continuously within the entire group of alkali elements.

However, having arrived at this stage, we have to admit that we are not sure about the tiny radius differences of our approach between the ions of K^+^ and Rb^+^ amounting to 1 pm, i.e., to 0.01 Å only. The measuring accuracy of surface tension measurement, although being high, cannot stand such a high requirement. Even the small difference of Δ*r*^+^_aq_ = 3 pm between the resulting value of the pseudo-alkali ion ammonium (NH_4_)^+^ and that of K^+^ could not be considered to be reliable if we took into account the measuring accuracy in connection with the standard errors given by the evaluation of the surface-excess data calculated from the adsorption isotherms in ref (2). Hence, at this stage, we could give the decreasing sequence of hydration radii as follows for sure:
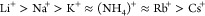
5

Concerning the hydration radius of Cs^+^, the following result gets noteworthy. First, if we excluded from the best-fit of eq 4 the data of cesium and then calculated its *r*^+^_aq_-value from the matching equation, a yet lower value of 232 pm (instead of 239 pm) would result. This finding underlines the fact that the hydration radius of the Cs^+^-ion is indeed noticeably smaller than those of Rb^+^ and K^+^. However, as we realize that the reliability of the measured π_cmc_-value is better than that of the corresponding *A*_min_-value calculated from the adsorption isotherm, we maintained the data of Cs^+^ for the final best-fit. The latter’s π_cmc_-value is distinctly higher than those of Rb^+^ and K^+^, suggesting a considerable geometrical difference between these hydrated ions. Actually, the corresponding σ_cmc_-value of Cs^+^-perfluorooctanoate represents the lowest surface tension value we have measured of an aqueous surfactant solution at all.

Second, whereas the absolute values of ref (3) and ours^2^ do not deviate from each other significantly; those of ref (4) are much larger, up to 44% for the smallest values of cesium.^3^ Third, it is worth mentioning that while the slope of d(*r*^+^_aq_)/dπ_cmc_ is roughly identical for refs (2) and (3), it becomes considerably weaker for the data of ref (4).

The resulting cations’ hydration radii are compiled in Table 1.

**Table 1:** Compilation of Alkali Ions’ Hydration Radii as Obtained from Various Sources

cation	Ads./cmc/naked ion radius^f^*r*^+^_aq_ [pm]	adsorption/σ_cmc_^e^*r*^+^_aq_ [pm]	Lunkenheimer et al.^d^*r*^+^_aq_ [pm]	Latscha et al.^c^*r*^+^_aq_ [pm]	Scheffer et al.^b^*r*^+^_aq_ [pm]	naked ion^a^*r*^+^ [pm]
Li^+^	**345**	344	342	340	382	60
Na^+^	**293**	300	305	276	358	95
K^+^	**260**	252	245	228	331	133
Rb^+^	**250**	253	248	232	329	148
Cs^+^	**239**	239	250	228	328	169
(NH_4_)^+^		256	245			

a Naked ion (crystal^6,7^).

b Scheffer/Schachtschabel; textbook.^4^

c Latscha, Kazmaier; textbook.^3^

d Adsorption isotherm at sub-micellar concentrations, eq 4.^2^

e Adsorption isotherm at sub-micellar concentrations including the parameter of σ_cmc_.

f Results obtained by calculating values of option e as a function of their naked ion radius^a^.

**Figure 5:**
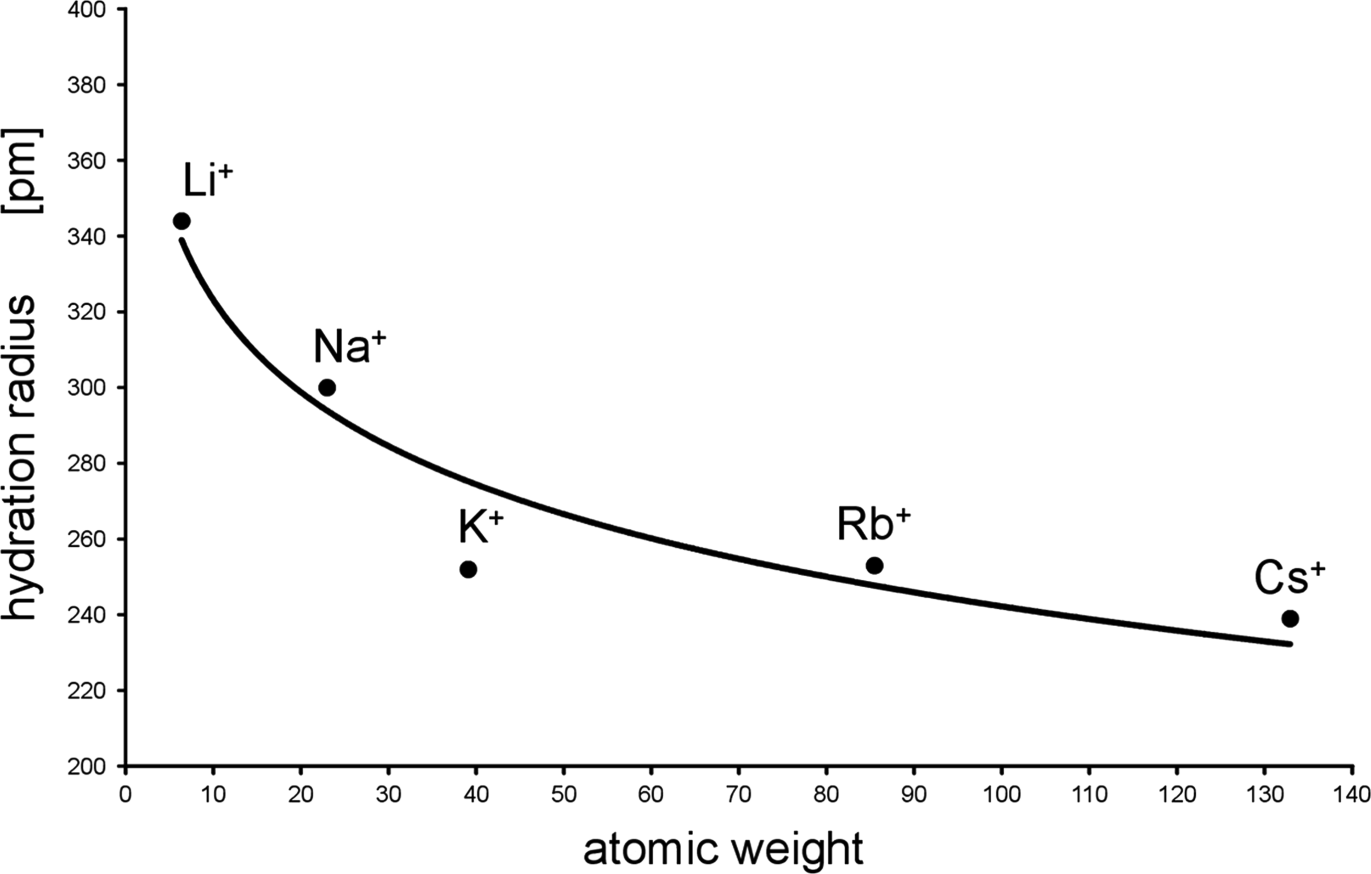
Alkali ions’ hydration radii calculated by eq 4 as a function of the related elements’ atomic weight.

Considering the findings contained in eq 5, the matter in question remains why the sizes of only two hydrated alkali ions, i.e., of potassium and rubidium, should be equal contrary to other chemical properties related being significantly different. With respect to experience in chemistry, this behavior seems a little odd. It may be due to the insufficient sensitivity of surface tension measurements, which is not enough to decide about possible small differences between the hydration radii of K^+^_aq_ and Rb^+^_aq_. One main result of our recent communication^2^ has been the conclusion that we do not need exceptional physicochemical assumptions to match the ionic amphiphiles’ adsorption properties adequately. To decide about the validity of these findings, we looked for a reliable reference state. We remember again Mendeleev’s Periodic System of the Elements.^5^ Mendeleev used the atomic weight as the reference state. If we do so by evaluating the ion’s hydration radius as a function of its atomic weight, we indeed obtain a successively decreasing hydration radius with increasing atomic weight. The best-fit applying the simple logarithmic equation *y* = *a* + *b* ln *x* results in a standard error of *r*^2^ = 0.94, possessing a maximal error of about 10%. Quite interestingly, all resulting *r*^+^_aq_-values become well discernible from each other. Thus, there is a noticeable difference in hydration radii of (K^+^)_aq_ and (Rb^+^)_aq_ of about 25 pm. This is illustrated in Figure 5.

Searching for the most reliable sequence of the alkali ions’ hydration radii, we take into consideration the data of the naked alkali ions obtained from X-ray investigations of solids (crystals).^6,7^ The sequence of the naked alkali ions’ radii well obeys Mendeleev’s law.

The naked alkali ions’ radii as a function of their atomic weight result in an excellent best-fit with a standard error of *r*^2^ = 0.998 using a quadratic function, the maximal error of which is about 3%. Assuming that the nature of hydration is dominated generally by equal electrostatic interaction for the chemically related alkali ions,^32−34^ there should exist some basic relationship between the naked alkali ions’ radii (crystals) and the related hydration extent of theirs. Thus, it is reasonable to evaluate our data on the ions’ hydration radii obtained by adsorption together with surface tension at micellization directly as a function of the naked ions’ radii.

By plotting tentatively the hydration radii as a function of the naked ions’ radii and applying the plain exponential function,
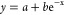
6provides the optimal best-fit characterized by a standard error of *r*^2^ = 0.987, *a* = 1.824, and *b* = 2.952. *y* stands for the hydration radii retrieved from our surface tension approach described above. *x* stands for the radii of crystal ions. It means high confidence. The maximal residual of hydration radii amounts to ∓3%. If we apply the simple quadratic function *y* = *a* + *bx*^2^, we get almost the same error of *r*^2^ = 0.984 with 99.9% confidence. The latter result underlines our suggestion that hydration should be related to the ion’s charge density mentioned above.

Thus, as a matter of fact, there indeed exists a reasonable relationship between the naked alkali ion radii and their hydrated ones in solution. It represents a continuously occurring decreasing sequence of the hydration radii of the alkali ions from lithium down to cesium.

Thus, using eq 6, we can even predict the radius of the hydrated francium ion to be about 2.31 Å.

Applying eq 6, we calculate the resulting values of ion hydration belonging to those of our surface tension approach. They are listed also in Table 1 (bold numerals).

As you can take from it, these hydration radii are practically identical to those of our approach except the two of the sodium and the potassium ions. Equation 6 tells us that it is not the hydration radius of the Rb^+^-ion that seems to be a little odd but it is the uncertainty in the *r*^+^_aq_-values of sodium together with that of potassium that have led us to a suggestion like this. Our surface tension approach resulted in hydration radii of about 1% too big for Na^+^_aq_ but of about 2% too low for K^+^_aq_. The alkali ions’ hydration radii obtained by this combined approach represent the most reliable ones that are well in line with Mendeleev’s law.^5^ Interestingly, the expected reasonable sequence of hydration radii comes out by it. Thus, we have to refine the sequence in eq 5 by the following ones, eq 7a,b:
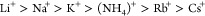
7a
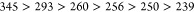
7bAll data are given in picometer.

All in all, we concluded that the hydration radii of the alkali cations contained in aqueous solution are not only in line with the fundamental properties of physical chemistry but also with Mendeleev’s law. In addition, we think that these values of the hydration radii are the most reliable ones so far determined.

Table 1 compiles the discussed data on ion hydration.^2−4^ The evaluation of surface tension investigations in connection with the data on naked ions of solid materials (crystals) has not only generally confirmed the former results but lead also to a refinement of the debatable equality of the *r*^+^_aq_-values of K^+^ and Rb^+^. This enables us to conclude that the data given by eq 7a,b are the true ones occurring in aqueous solution. This result is well compatible with other physico-chemical properties, such as atomic volume,^7^ thermodynamics, ion exchange, spectroscopic ones (see below), which reveals a clear sequence of changes of properties of the heavier ions K^+^-, Rb^+^-, and Cs^+^-ions too.^35,36,38−42^ The chemical analogy of the alkali ions is confirmed by the same cubic, body-centered crystal structure for every ion.^7^ This achievement in improving the sequence of the alkali ions’ hydration properties quantitatively requires the extension our model on the arrangement of counterions in the amphiphiles’ boundary layer as presented schematically by Lunkenheimer et al. in Figure 7 in ref (2).

**Figure 6:**
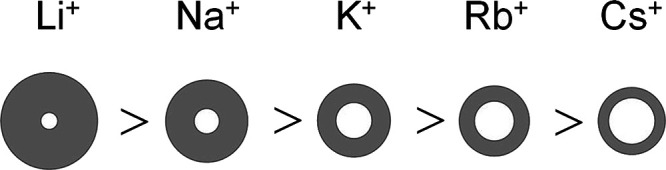
General framework of area occupancy of the alkali cation in the boundary layer. Ratios are given approximately in scale. Filled circles represent the space of hydration sphere. Empty circles denote the space of naked ions. For the minimal cross-sectional area of the entire ionic alkali-perfluorooctanoate molecule, each is exclusively determined by the size of its hydrated cation; the anion’s cross-sectional area remains hidden. (This is in contrast to Figure 7 in ref (2).)

The basic principle of it remains valid stating that the surfactant’s cross-sectional area will exclusively be determined by the size of counterions. However, in our last communication,^2^ we still assumed in accord with refs (3) and (4) that the sizes of the heaviest hydrated ions (K^+^)_aq_, (Rb^+^)_aq_, and (Cs^+^)_aq_ are practically identical. Unlike this assumption, we now succeeded in proving that these three ions’ size does yet further continuously decrease. Thus, the adsorbed alkali perfluorooctanoate surfactant of the smallest surface cross-sectional area, i.e., that of cesium, is still determined exclusively by its hydrated counterion’s size. Hence, we have to refine the basic principle of the counterion effect derived in ref (2) by extending it to the entire group of alkali ions. It means that, generally, the minimal cross-sectional area occupied by any adsorbed alkali perfluorooctanoate will exclusively be determined by the size of the counterion bound stoichiometrically to the perfluorooctanoate anion. Equation 7a represents a general law valid under the required conditions of the pseudo-nonionic nature of the ionic surfactants and of surface-chemical purity of their solutions. Thus, the refined generalized pattern of the schematic in Figure 7 of ref (2) is to be substituted by Figure 6 of this communication.

In it, the cross-sectional area of the hydrated alkali ions is illustrated together with the size of the naked ion (inner sphere) and its hydration shell. By this drawing, the pattern of hydration appears especially convincing. As we know now, that the hydration shell of the counterion decreases successively down to the heaviest cesium ion, it is reasonable to assume that there will remain a small shell of hydrated water molecules left to it likewise according to ref (36).

Estimating these results on the background of the voluminous investigations on the hydration of alkali and alkali earth metal ions in aqueous solution,^36−50,52,59−65^ we have to realize that their results have so far been dissatisfied. Although modern methods are applied such as nuclear magnetic resonance spectroscopy (NMR),^36,43−45^ X-ray diffraction,^11,46^ ionization spectroscopy,^47^ Raman,^37^ infrared,^38^ and mass spectroscopies,^48^ and calorimetric,^39^ cation-exchange,^40,49,50^ as well as electro-catalysis studies,^51^ we do not get concrete, reliable information on the real nature of the hydration sphere. In no case we found established details on the fundamental question of what will be the effective size of the hydrated cation in aqueous solution.

Furthermore, the solutions’ concentrations of the alkali ions applied for these complicated methods of investigation are often extremely high, aiming to retrieve an adequate quantity of the measuring signal, usually of concentrations between 1 and 10 M,^43,45,52^ which are hardly met under conditions of application. Surfactants are usually applied at much lower concentrations between 10^–4^ M > *c* > 10^–1^ M. Hence, it seems incomprehensible that we retrieved correct values on the ions’ hydration by surface tension measurements of solutions the bulk concentrations of which are a few orders of magnitude lower. However, at this state of investigation, we would like to remind you that its main aim was to get true information on the nature of the ionic surfactants’ adsorption. In it, the most important adsorption parameter is surface excess, in particular surface excess at saturation Γ_∞_. Realizing that this parameter represents the surfactant concentration in the boundary layer and that the latter consists of an extremely small thickness, we can roughly calculate the surface excess by its corresponding three-dimensional bulk concentration. Assuming the thickness of the boundary layer of say δ ≅ 100 Å = 10^–6^ cm and an average value of Γ_∞_ ≅ 5 × 10^–10^ mol/cm^2^, we get a bulk surface concentration of Γ_∞_/δ = 0.0005 mol/cm^3^ corresponding to 0.5 M. In the case in which we assume that δ ≅ 10 Å = 10^–7^ cm, the corresponding surface concentration at saturation would amount to 5 M. Although this represents a rough estimation only, it clearly explains why surface tension is so sensitively related to the adsorbent’s properties. The optimal condition to observe any molecular characteristic of the adsorbed surfactant is that of adsorption saturation. Now, with respect to the results of our new model of the ionic surfactants’ adsorption put forward in ref (2), it means that any measurable difference in the σ_cmc_-values will necessarily be attributed to the change of the related counterion in the adsorption layer. Consequently, the range of our approach is not restricted to the lighter alkali counterions, but it does comprehend the entire group of alkali ions from lithium up to cesium. This result represents a distinct improvement of the model of the ionic surfactants’ adsorption.

All in all, our endeavor to model the adsorption of ionic amphiphiles appropriately has not only solved the apparent disagreement of size between lighter and heavier alkali ions but also has yielded a kind of valuable “scientific byproduct”, i.e., the true values of hydrated counterions.

Exact knowledge about ion hydration is especially important for understanding the molecular basis of selectivity of biological and artificial membranes.^46,53,54^ Membrane proteins may work as selective ion channels, in particular for Li^+^-, Na^+^-, and K^+^-ions having multiple functions in the biological cells.^46,54^ The efficacy of lithium salts for the treatment of manic, depressive psychosis in man is now well accepted. However, its clinical action is far from being its only biological effect. Lithium effects interfer with a variety of biological effects and occur essentially within all classes of organisms from higher plants to viruses.^55^ K^+^-channel gating is a well-known phenomenon important, for example, in neuronal and cardiac pacemaker cells.^54^ However, originating from our findings of this investigation, we are confronted with a few odd results. Thus, for example, in ref (46), constraining hydrated lithium or sodium in narrow pores of radii between 1.5 and 2.5 Å is discussed. Inside a narrow pore of 1.5 Å, the cost of constraining a hydrated potassium ion should be smaller than that of hydrated sodium. However, the opposite should be true for pores of radius about 2.5 Å. The reason is discussed in terms of different distortions of the ions’ hydration shell. The authors discuss the water molecule’s arrangement around the ion up to a third hydration shell. We cannot understand how pore radii as small as 1.5 Å should enable a hydrated ion to pass provided that the passage is due to geometrical constraints.^46,53,54^ The size of all hydrated alkali ions is roughly twice the radius of 1.5 Å.

The much better fit based on our conclusions from investigations on artificial membranes was compared.^56^ Samec et al. evaluated the alkali ions’ diffusion coefficients (Li, Na, K, Rb, and Cs) from ion-exchange membrane measurements. They conclude that the alkali metal ions’ diffusion through the membrane reflects mainly a steric effect without changing the mechanism of transport in bulk water. A recent communication on highly selective separation and recovery of Li(I) from Na(I) and K(I) using a polymer inclusion membrane reports on separation factors well in line with our conclusions on the importance of the ions’ geometry.^53^ Recently, the importance of the effective radii of alkali ions has also arrived at the field of application, such as advanced materials. In ref (49), the effect of ion radius on the efficiency of Na ion storage for a sodium ion battery is reported. The comprehensive review article of Xu et al.^57^ reveals why any gain in knowledge on the feature of the lithium- and sodium-ions is of utmost importance for improving the high-energy storage coming from renewable sources. Li^+^- and Na^+^-ion batteries are a contender among the various available electrochemical storage technologies based on batteries because of the much higher energy stored per unit weight compared to other conventional batteries. However, how little is known on the genuine mechanism of ion hydration you may take from a contribution on Li^+^-conducting mediators for enhanced electrochemical performance in which the authors speculate loosely on different options of hydration within the same solution by hypothetic schematics. Therein, the sodium ions are bound to two or three water molecules in the bulk but are partially dehydrated at the boundary layer close to the electrode surface, respectively.^58^ This is contrary to our results reported in this and our previous contributions.^1,2^ It is chemically irrational.

The endeavor to investigate the nature of the cations’ hydration is accompanied by quite a few theoretical ones on molecular dynamics simulations applying various options.^52,63,64,67,34−36,60−62^ These approaches may simulate the nature of hydration by applying quite a lot of molecular properties such as the hydration structure,^52^ partition function ratio,^50^ dipole moment,^59^ residence time, self-diffusion coefficients and orientation^60^ of water molecules,^47,61,62^ electrostatic effects,^42^ angle of dipoles,^59^ distribution sphere around the cation, M–O bond distance,^35,41^ and flexibility within the hydration shell.^36^ In most cases, communications provide the average number of hydrate water molecules around the cation. However, as shown already on the details of which number will be effective in reality, there is no agreement. Usually, the strongest hydrated cation Li^+^ is assumed to possess six or seven water molecules, whereas the less hydrated ones Rb^+^ and Cs^+^ are estimated to have only two to four. Furthermore, there are also works contradicting this conclusion by stating the inverse, namely, that the effective (stable) hydration number of the lithium ion will be rather 4 instead of 6^11,43,50,63^ or that those of Rb^+^ and Cs^+^ are 8 and 10^35,41^ or 7 for the Rb^+^-ion’s first hydration shell.^64^

The most disappointing conclusion of ours is that obviously it has, so far, not been possible to clearly establish whether there will be a discrete effective, stable hydration shell at all, or whether there will be an additional second one, the nature of which has to be discriminated from the former.^35,36,38,62^ These studies strive to find out the thermodynamically most stable hydration number.^42,62,65^ Unfortunately, however, even if you really knew the true hydration number you would not necessarily retrieve the effective radius of the cation’s hydration shell in the solution concerned.

Summarizing the above discussed contradictory results, we conclude that any information on the ions’ hydration nature will only be true if it is proved to be well founded by appropriate, reliable experiments. For we have done so, we are able to give reliable quantitative data on the alkali ions’ hydration radii in aqueous solution. In addition, we can provide quantitative information on the effective radius of hydration of the pseudo-alkali ammonia cation.

The main result consists of the fact that we are now able to exactly discriminate between the hydration radii of the different alkali ions effective in solution.

The observed successive decrease of hydration radii given in eq 7a,b is in line with some studies on various physico-chemical properties of the alkali ions, such as calorimetric,^39^ resin exchange,^40,49^ and ion channel selectivity^50^ ones. Yang wrote that “the effective radii and structures of hydrated Na^+^, K^+^, Rb^+^, and Cs^+^ play a key role in ion transport properties, such as ion channel selectivity”.^50^ Our findings also prove that the ammonia cation’s (NH_4_)^+^ hydration shell size is indeed similar to that of the alkali cation ones as is said by textbooks on inorganic chemistry.^8^ Its geometrical size is in between the hydration radii of the potassium and rubidium cations.

## Conclusions

1. Progressing from our results in ref (1), we provided evidence that the pseudo-nonionic 1:1 extended chain amphiphiles’ surface excess at saturation Γ_∞_ will exclusively be determined by the alkali counterions’ dimension.^2^ However, this was apparently limited to the three lightest alkali cations of lithium, sodium, and potassium because the two heaviest ions of rubidium and cesium were seemingly of equal size, namely, like that of potassium. The resulting discrepancy remained chemically inconsistent insofar as the surfactant adsorption of the lightest counterions would thus be exclusively determined by the counterion, whereas it would become negligible for the heavier counterions.
2. The results of modern methods of investigating the hydration of alkali and alkali earth metal ions in aqueous solution^36−50,52,59−65^ have so far been dissatisfying. They do not provide concrete, reliable information on the real nature of the hydration sphere. Furthermore, these methods need to apply bulk concentrations greater by 2 or 3 orders of magnitude than those of the surfactant solutions applied in this investigation to retrieve an adequate quantity of the measuring signal. Having taken into account the latter findings, we realized that the surfactant’s concentration in the boundary layer constituted as a three-dimensional analog will well meet the conditions of very high concentrations. In particular, the condition of surface saturation should be favorable to retrieve molecular information, provided there is a physical quantity that is related to the surfactant’s surface excess. Fortunately, surface tension measurement will do so.
3. Therefore, we have included surface tension measurements by additionally exploiting surface tension of their micellar solutions. Interestingly, we found that the equilibrium surface tension values of 1:1 pseudo-nonionic surfactant solutions at their critical concentration of micelle formation, σ_cmc_, represent a characteristic parameter of adsorption and micellization. Their value is constant independent of concentration at *c* ≥ cmc, provided the required thermodynamic conditions, in particular that of surface-chemical purity, are obeyed. Hence, following our novel model, the counterions’ size in the boundary layer at the air/water interface can quantitatively be calculated by exploiting the related parameter σ_cmc_. This has led to a distinct improvement of our model. It means that the principle of counterion binding within the adsorption layer put forward in ref (2) not only remains valid throughout the entire group of the alkali ions but it additionally enables us to determine the size of the hydrated counterions. In any case, the ionic surfactant’s cross-sectional area will be determined exclusively by the size of its hydrated counterion, cf. Figure 6. This conclusion represents a general principle of adsorption of pseudo-nonionic extended chain surfactants. Only in the case in which the amphiphile’s hydrophobic part gets distinctly branched, it will determine the entire molecule’s surface area demand instead of the counterion.^66^
4. Our results support the discreteness of the hydration radii in solution. They underline investigations that favor stronger hydration for lighter alkali ions.^60,67,68^ As a matter of fact, these results are related to ionic surfactants fulfilling the conditions of pseudo-nonionic behavior met for medium surface activity.^2^ Although we so far have provided experimental evidence for cationic counterions only, we guess that anionic counterions will obey analogously in adsorption. Nevertheless, this has to be proven by experiments.
  These findings culminate in the simple conclusion that we must not apply strange assumptions on an exceptional water structure in the boundary and/or in the ions’ sphere of hydration to better understand the role of counterions in adsorption and micellization. There will be no distinction between the counterion’s hydration behavior in bulk, in the double layer, in the adsorption layer, and at the micellar interface, by it going in line with basic assumption of inorganic chemistry and supporting the conclusions of refs (32) and (33) on counterion binding to ionic micelles.
5. Taking up Mendeleev’s thoughts on the periodical dependence of the chemical elements’ properties on their atomic weight and applying them to the investigation of surface tension has led to a further refinement of the sequence of hydration radii of the alkali ions. Thus, so to speak as a “scientific byproduct”, we became able to provide the most reliable data on the entire alkali cations’ hydration available so far (cf. Table 1).These findings are important not only for adsorption but also for related fields reaching from the environment or energy storage to virology. In addition, they should be of high interest for all kinds of molecular dynamics simulations on the structure of alkali metal ions’ hydration.
6. We would like to address some general conclusions on the experimental prerequisites of interfacial research. There is, first of all, the importance of adequate purity in investigating the fundamentals of surfactant adsorption and micellization. It is hardly understandable that basic surfactant properties like those reported on here on the effects of counterions have been inaccurate until now, in spite of various modern high-resolution methods of research. Years ago, Prosser and Franses put forward an estimating review on the quality of research of ionic surfactants at the air–water interface. Their conclusion stated unequivocally that the majority of the related experimental investigation is inadequate.^69^ From our point of view, it is inconvenient that the discussed communications have usually used sodium dodecylsulfate (SDS) as a standard surfactant, which is especially plagued with very effective surface-active contaminants when applied “as received”.^70^ Concerning reliability of experimental results on surface tension of micellar solutions, even the date of issue cannot be used as a criterion of reliability. With respect to this investigation, one of the oldest references appears to be the most reliable one.^18^ It is interesting to note that we found a very old reference published about one century ago in which the relative hydrations of various ions, including the alkali ones, have been calculated by diffusion quite distinctly in the correct sequence like
  Li^+^ > Na^+^ > K^+^ > (NH_4_)^+^ > Rb^+^ > Cs^+^^68^
  Second, you should try to find out convenient molecular model structures.
  Researchers investigating surface properties need to realize that an adsorbed amphiphile requires a particular grade of purity that cannot be obtained by purity methods based on bulk properties. With respect to the measuring method applied, it is not its kind that is important but the requirement that the measuring signal is retrieved from the adsorption layer itself.
7. This contribution clarifies the discussion on a fundamental problem of chemistry that has still been fairly confusing.
